# Separation of Teeth

**Published:** 1896-02

**Authors:** 


### Separation of Teeth.

Rubber for separating the teeth is little used on account of its great activity and of its disposition to work its way toward the neck of the tooth, thereby pressing on the gum and causing considerable pain. Yet rubber is made in special forms and used for this purpose still. This style of rubber often gets quite stiff, hard and rotten, making it, when wanted, unfit for use. Rubber-dam is always at hand, is always fresh and always ready for use. If a piece of this be *twisted in a roll between the thumb and fingers* it can be made in any size necessary for the case in hand, and being thus made cylindrical, is in the best form for application. Three, four, five or a dozen turns can be made of a small discarded piece of an inch square to place between the teeth to effect their separation.—*British Journal of Dental Science.*

